# Towards the endgame and beyond: complexities and challenges for the elimination of infectious diseases

**DOI:** 10.1098/rstb.2012.0137

**Published:** 2013-08-05

**Authors:** Petra Klepac, C. Jessica E. Metcalf, Angela R. McLean, Katie Hampson

**Affiliations:** 1 Department of Applied Mathematics and Theoretical Physics, University of Cambridge, Wilberforce Road, Cambridge CB3 0WA, UK; 2 Department of Zoology, University of Oxford, South Parks Road, Oxford OX13 PS, UK; 3 Institute of Biodiversity, Animal Health and Comparative Medicine, University of Glasgow, Glasgow G12 8QQ, UK

**Keywords:** immune escape, susceptible build-up, vaccine refusal, vaccination coverage

## Abstract

Successful control measures have interrupted the local transmission of human infectious diseases such as measles, malaria and polio, and saved and improved billions of lives. Similarly, control efforts have massively reduced the incidence of many infectious diseases of animals, such as rabies and rinderpest, with positive benefits for human health and livelihoods across the globe. However, disease elimination has proven an elusive goal, with only one human and one animal pathogen globally eradicated. As elimination targets expand to regional and even global levels, hurdles may emerge within the endgame when infections are circulating at very low levels, turning the last mile of these public health marathons into the longest mile. In this theme issue, we bring together recurring challenges that emerge as we move towards elimination, highlighting the unanticipated consequences of particular ecologies and pathologies of infection, and approaches to their management.

## 1. Introduction

Control measures have led to tremendous reductions in the incidence of infectious diseases that affect human and animal health. Vaccination has successfully interrupted circulation of poliomyelitis, measles, rubella and has drastically reduced the incidence of foot-and-mouth disease (FMD) and canine rabies throughout the Americas; mass drug administration has significantly reduced the transmission of lymphatic filariasis and onchocerciasis (river blindness) across their endemic ranges, whereas the number of cases of dracunculiasis (guinea worm) has fallen by more than 99 per cent since 1986 through behavioural interventions without the use of either drugs or vaccines.

**Figure 1.**
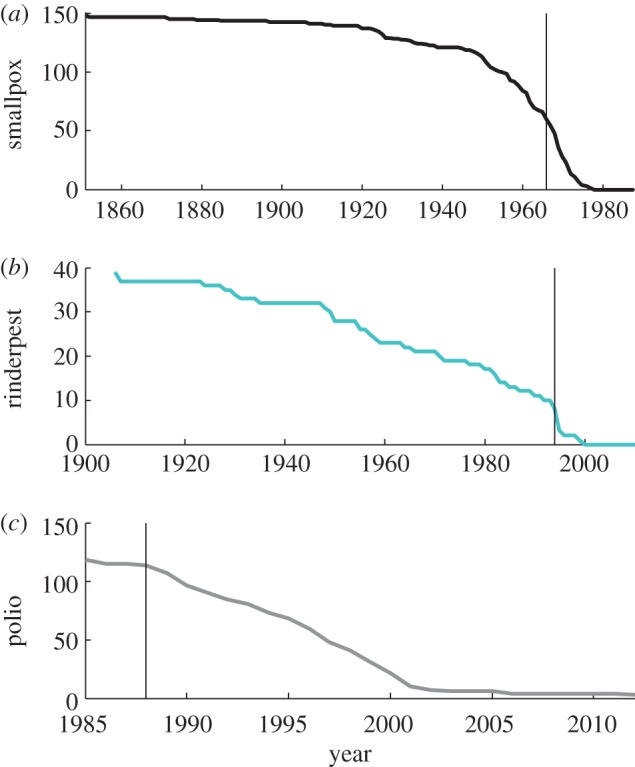
The number of countries endemic for (*a*) smallpox, (*b*) rinderpest and (*c*) polio. Vertical line indicates the beginning of global eradication initiative for respective diseases. (Online version in colour.)

However, despite these successes, eradication *per se* of established pathogens has been limited. Only one human (smallpox) and one animal (rinderpest) disease have been eradicated to date.^1^ Both diseases were perceived as a major threat and vaccination, the key intervention to interrupt their transmission, was a priority long before the initiation of global eradication campaigns (figure 1). Thermostable vaccines were developed for both viruses, eliminating the need for a refrigerated supply chain (‘cold-chain’), and vaccines were kept to a high standard, monitored by independent quality-control centres [1,2].

The smallpox and rinderpest programmes built on the experiences of previous failed efforts to eradicate hookworm (1907), yellow fever (1915), yaws (1955) and malaria (1955) [3]. Each of these ultimately unsuccessful efforts provided valuable lessons. Mass treatment for hookworm did not cure, and therefore it only decreased infection intensity. The existence of an animal reservoir in nonhuman primates hampered yellow fever eradication [4]. The importance of large-scale pilot programmes ahead of the eradication effort, ongoing research in parallel to the programme and intensified surveillance during the endgame became evident after the failure to eradicate yaws,^2^ via an effort that lasted from 1954 to 1967 when treatment with injectable long-acting penicillin was prematurely discontinued. The Global Malaria Eradication Programme (1955–1969) was primarily defeated by the emergence of drug resistance and resistance of mosquito vectors to insecticides, and was further hindered by high costs (billions of US dollars even then) [6]. The structure of the programme also proved too rigid for the different regional and local requirements of malaria-endemic areas, and suffered from a lack of research and community involvement [3,6,7]. By contrast, smallpox and rinderpest campaigns were flexible, and relied on the involvement of local communities [8], with strategies being constantly refined in response to ongoing research and field studies [2,7,9].

The success of the smallpox and rinderpest campaigns was also a function of the biology of these pathogens; and the diversity of causes underlying the failures of historic eradication programmes reflects the diversity of the relevant pathogens’ biology and population dynamics, and specifically how these dynamics were affected during the last mile of elimination, or the endgame.

**Figure 2.**
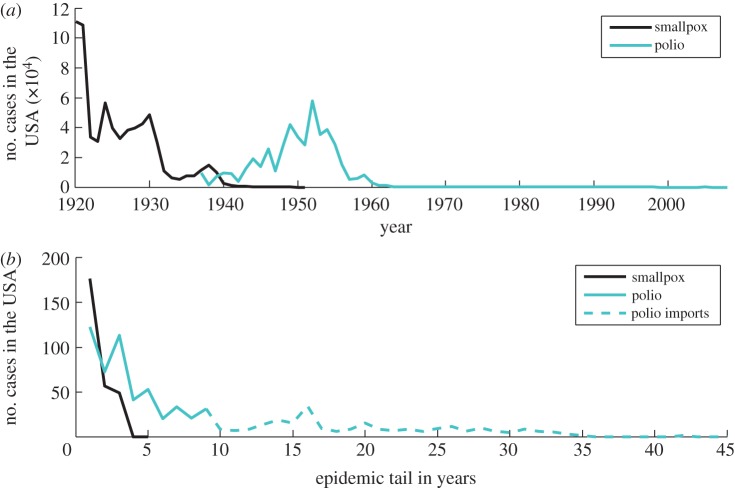
Smallpox and polio elimination in the USA—the total number of cases. (*a*) Total reported number of smallpox and polio cases per year in the USA. (*b*) Epidemic tail for smallpox and polio in the USA in years. Epidemic tail—years with less than 200 reported cases. (Online version in colour.)

We use the term ‘endgame’ to refer to the final stages of an elimination or eradication programme, when disease is still circulating, although at much reduced levels (i.e. during the epidemic tail illustrated in figures 1 and 2). For any disease targeted for elimination, international organizations such as the World Health Organization (WHO), World Organization for Animal Health (Office International des Epizooties, OIE) and the Food and Agriculture Organization (FAO) generally provide guidance and/or a regulatory framework with epidemiological or programmatic milestones: the progressive control pathway for FMD is one such example [10]; another is the pathway defined for malaria elimination (http://www.who.int/malaria/publications/atoz/9789241596084/en/index.html). While such operational definitions are clearly helpful for specific pathogens, for the multi-pathogen perspective taken here, we define ‘endgame’ more conceptually. Specifically, we take it to refer to the stage in the elimination programmes when the goal seems in sight, and operational targets typically become time-limited, because the intensity of effort required is too great to be maintained indefinitely.

New and often unforeseen obstacles arise during the endgame (see box 1 for an overview of endgame challenges for different pathogens detailed in this theme issue). These may be biological, operational, technical, economic or political in nature. With this in mind, we solicited articles for this special issue from a diverse range of authors covering these perspectives. Here, we introduce why the endgame differs from the ‘middle game’ (or period where interventions are sufficiently intense to control disease to some degree, but not sufficient to dramatically change the ecology, or surveillance and control needs) across pathogens that are currently targeted for local or regional elimination or even eradication (see box 2 for definitions). We list major knowledge gaps across diseases, outline some of the current issues in implementation of control methods and finally, conclude by discussing prospects for success.

Box 1. **Pathogens targeted for regional or global elimination covered in this special issue.**

**Dracunculiasis**. The first parasitic disease targeted for eradication, dracunculiasis (guinea worm) is caused by the parasite *Dracunculus medinensis* following ingestion of water contaminated with larvae-harbouring copepods. Worms up to 1 m long begin to emerge from infected people a year after drinking contaminated water. The number of cases fell from 3.5 million in 1986 to 542 in 2012 [11] without the use of a vaccine or medical treatment—control efforts focus on providing non-contaminated drinking water, vector control, community education and involvement supplemented by active surveillance [12]. Political instability in the areas of Mali and South Sudan presents the biggest endgame challenge for eradication. The estimated cost for the programme over three decades is $350 million [13].

**Foot-and-mouth disease.** FMD is a highly transmissible viral disease of cloven-hoofed animals, particularly cattle, sheep, pigs, goats and deer, and is associated with tremendous economic losses. It is caused by an aphthovirus of the Picornaviridae family that has seven different serotypes (O, A, C, SAT 1, SAT 2, SAT 3 and Asia 1); infection with one serotype does not confer immunity against other serotypes. Vaccination protects from the disease, but does not prevent infection or carriage, so certain regions prohibit prophylactic vaccination against FMD (e.g. the European Union) complicating trade. Control methods focus on culling of infected and in-contact herds and strengthened border controls to prevent introductions from endemic areas through regional movement of animals and their products. In South America, systematic mass vaccination is being used to regionally eliminate infection [14]. Endgame challenges include maintaining high coverage, political commitment and financial support, detecting carriers and confirming the change in status from disease-free with vaccination to free without vaccination, as withdrawing vaccine prematurely can lead to re-emergence.

**Lymphatic filariasis**. Also known as elephantiasis, this disfiguring and debilitating disease is characterized by swelling of the limbs and genital organs, kidney damage and painful swelling of lymph nodes. Lymphatic filariasis is caused by filarial nematodes *Wuchereria bancrofti* (90% of all cases), *Brugia malayi* and *Brugia timori* that are transmitted to humans through bites of infected mosquitoes. Transmission can be successfully interrupted by the mass administration of donated drugs ivermectin and albendazole, or with diethylcarbamazine and albendazole. Most cases are in India, Indonesia and Nigeria [13]. Inadequate political and financial support to scale-up mass drug administration, contraindications with the parasite *Loa loa* that can cause fatal side-effects upon treatment, and political insecurity in certain areas poses the main challenges [15].

**Malaria**. Malaria is a mosquito-borne disease caused by four different protists of the *Plasmodium* genus that multiply in red blood cells of infected humans, travel to the liver to mature and later reproduce in infected mosquitoes, ending the life cycle. Malaria still takes a life of an African child every minute, and causes over 200 million infections a year [16], reinforcing the poverty of sub-Saharan African countries through effects on fertility and population growth, productivity, savings and investment, premature mortality and medical costs [17–19]. After a hiatus of over four decades, malaria elimination is back on the global health agenda, inspired by the Roll Back Malaria Initiative launched by WHO in 1998 and the Gates Malaria Forum in 2007. Main challenges include drug resistance and resistance of mosquitoes to insecticides. Contrary to popular belief, stopping post-elimination transmission from imported cases might not pose a great challenge as there is evidence that local elimination is surprisingly stable [20].

**Measles**. A highly contagious childhood viral infection, measles remains one of the leading causes of death of children under 5 years of age despite the existence of an affordable and effective vaccine. Approximately 158 000 people died of measles in 2010; more than 95% of those were in low-income countries with weak health systems [21]. Since its establishment in 1974, WHO's Extended Programme on Immunization (EPI) has been the main tool and driver of childhood immunization against several diseases including measles. More recently, spurred by the millenium development goals (MDG) to reduce childhood mortality, the Measles and Rubella Initiative has led to a 71% reduction in measles deaths from 1990 to 2011. Main challenges include the build-up of susceptibles that are repeatedly missed by campaigns, the need to tailor control strategies to specific demographic and public health contexts, and vaccine refusal in the developed world where incidence has been very low for decades [22,23].

**Meningococcal meningitis**. Several bacteria can cause meningitis—a transmissible severe inflammation of the meninges, or the protective lining around the brain and spinal cord—but *Neisseria meningitidis* is the one that causes large outbreaks in the meningitis belt of sub-Saharan Africa. If untreated, it is fatal in half of all cases. Of the twelve serotypes of *N. meningitidis*, six are epidemic (A, B, C, W135, X and Y). Conjugate polysaccharide vaccines are available for four serotypes (A, C, W135, Y); for serotype B polysaccharide vaccines cannot be developed due to antigenic similarity with a polysaccharide in human neurological tissues (vaccines based on outer membrane proteins are used instead; WHO fact sheet). In 2010 and 2011 more than 55 million persons aged 1 to 29 years in Burkina Faso, Mali and Niger received conjugate vaccine that reduces carriage and transmission. It is thought that high coverage in this age group can eliminate meningococcal A epidemics in sub-Saharan Africa, but the main challenges are achieving and maintaining vaccine coverage [24].

**Onchocerciasis**. Also known as river blindness, onchocerciasis is caused by a parasite *Onchocerca vulvus* that is transmitted to humans by bites of *Simulium* flies and is the world's second-leading infectious cause of blindness (after trachoma). Microfilariae migrate to the skin and eyes, and after the nematode dies, their endosymbiont *Wolbachia pipientis* is released and causes a severe inflammatory response by the human immune system, leading to itching and sometimes blindness. Transmission can be interrupted by long-term mass administration of ivermectin; a minimum of 15 years of annual treatment is required, which is challenging especially in remote populations with limited access. Severe adverse effects to treatment in patients also infected by *Loa loa*, are an additional complication [15]. Emerging ivermectin resistance in *O. vulvus* may be an additional obstacle [25,26].

**Poliomyelitis**. Polio mainly affects children under 5 years of age and leads to irreversible paralysis in one in 200 infections [27]. Since the beginning of the Global Polio Eradication Initiative (GPEI) in 1988, polio cases have decreased by over 99% and the number of polio-endemic countries has been reduced from over a hundred to three (figure 1): Afghanistan, Pakistan and Nigeria. Challenges to polio eradication include the need for multiple doses for sero-conversion, reduced efficacy of oral polio vaccine in areas with high prevalence of enteric infections, gaps in serotype-specific immunity following local interruption of transmission, vaccine refusal and circulating vaccine-derived viruses [28], as well as political instability and growing danger to health workers [29]. The total cost for this effort currently exceeds $9 billion [30].

**Rabies**. An almost invariably fatal zoonotic disease that affects the central nervous system, the virus is commonly transmitted by bites of infected animals. Globally, different regions are at different stages of rabies control and elimination. Canine rabies, the main cause of humans rabies deaths globally, has been reduced by greater than 90 per cent in Latin America and the Caribbean due to mass dog vaccination [31]. High dog population turnover means that repeat vaccination campaigns are needed to sufficiently maintain coverage for transmission to be interrupted. In the Americas, achieving and sustaining high coverage remains a challenge in the poorest populations, which are the last strongholds of infection. In Europe, mass vaccination eliminated dog rabies in the mid-twentieth century (except for Turkey), at which point a virus variant circulating in fox populations became apparent. Oral rabies vaccination has since eliminated fox rabies from western Europe, challenging the notion that infection cannot be eliminated from a wildlife reservoir. A cordon sanitaire is now necessary to prevent incursions from neighbouring endemic areas re-seeding infection [32].

Box 2. **Definitions.**

*Eradication*: permanent reduction to zero of the worldwide incidence of infection caused by a specific pathogen established in a human or animal population, as a result of deliberate efforts, with no more risk of reintroduction.

*Elimination* (interruption of transmission): reduction to zero incidence of infection caused by a specific established pathogen in a defined geographical area, as a result of deliberate efforts; continued actions to prevent re-establishment of transmission may be required.

*Control*: reduction of disease incidence, prevalence, morbidity and/or mortality to a locally acceptable level as a result of deliberate efforts; continued intervention measures are required to maintain the reduction.

*Elimination as a public health issue*: targets set for political reasons, dependent on diseases and goals, for example, less than one case in 10 000 for leprosy [33].

*Basic reproductive number*, *R*_0_: the average number of secondary cases caused by a single infected individual in a completely susceptible population.

*Effective reproductive number*, *R*_E_: the average number of secondary cases caused by a single infected individual in a partially immune population (or where proportion *p* of the population is receiving control, *R*_C_ as in Smith *et al*. [20]); *R*_E_ = *R*_0_(1 − *p*). Determines how quickly we reach elimination; for *R*_E_ = 0.75 time to elimination is twice as long as for *R*_E_ = 0.5 if all other things were equal [34].

*Herd immunity*: the level of population-specific immunity where the pathogen is unable to maintain its reproductive rate above unity and continue circulating in the population; crucial for eradication efforts, as it is not necessary to vaccinate everyone in order to eliminate a pathogen.

*Critical eradication threshold*, *p*_c_: the proportion of the susceptible population that needs to be vaccinated in order to interrupt transmission in the population and achieve herd immunity; *p*_c_ = 1 – 1/*R*_0_.

## 2. Why does the endgame differ from the middle game?

### (a) Differing epidemiology

#### (i) Susceptible build-up

When infection is no longer circulating, or circulating at very low levels, the epidemiology of the target pathogen may change. For immunizing infections, the number of susceptible individuals in the population may increase during the ‘honeymoon period’ of low incidence (and consequently low transmission) following mass vaccination campaigns [35], or following local extinction. Both phenomena may result in huge outbreaks after susceptible numbers reach a threshold level, or the infection is reintroduced into an area where it had previously been eliminated. Massive outbreaks have repeatedly been observed for measles, for example in Mongolia in 2001 [36] and more recently in Burkina Faso in 2009 [37], illustrating that effective interventions short of eradication can lead to problematic short-term outcomes even if immunity is lifelong. Perceived risk also falls because cases are rare, but this is a fallacy; the effective reproductive number (*R*_E_; box 2), increases owing to the accumulation of susceptibles, thereby increasing the risk of a major outbreak.

#### (ii) Increase in the age of infection

Following the implementation of childhood vaccination campaigns, the vast majority of young individuals are protected, which can drive up the average age of infection. The measles outbreaks described above, that occur in contexts where susceptibles have accumulated via a lack of large outbreaks, and incomplete vaccine coverage, have much higher incidence in older age groups. Increased age of infection can have negative ramifications. It can increase the burden and the costs of infection (e.g. by increasing the number of cases of congenital rubella syndrome [38]) or complicate surveillance, as school-age or younger children are typically easiest to locate.

#### (iii) Waning of immunity

For immunizing infections where immunity needs boosting, other issues may arise. Lack of natural boosting contributes to waning of immunity in the case of pertussis (also known as whooping cough) and this can lead to resurgence in incidence despite high vaccination coverage [39,40]. Such resurgence in Western countries poses a particular, life-threatening risk for unvaccinated infants [41].

#### (iv) Pathogen change

Prolonged use of treatment or prevention measures can additionally generate selection pressure that can lead to pathogen adaptation, antigenic divergence or vaccine escape [42]. There is evidence that such adaptations have contributed to decreased vaccine efficacy and waning of immunity for pertussis [40,43], whereas some polio vaccine strains reverted to virulence by recombination with another virus (C-cluster coxsackie A viruses) [44]. As the main pathogen strains decline, minor strains may become more important—such as polio type 1 and 3 after eradication of type 2, *variola minor* in the case of smallpox and the mild forms of rinderpest that circulated in wildlife populations.

#### (v) Atypical infection reservoirs

Once common sources of infection are controlled, other, sometimes atypical, sources become important. For polio, asymptomatic and immunocompromised individuals who excrete the virus long-term will continue to cause new infections [45]. For measles, the decrease in perceived individual risk of disease contributes to vaccine refusal, leading to outbreaks in unvaccinated subpopulations [22,46]. During the rinderpest endgame, seasonally migrating pastoralists’ herds repeatedly spread the disease to adjacent sedentary livestock [2]. For treatable neglected tropical diseases (NTDs), most drugs used in preventative chemotherapy and mass drug administration have exclusion criteria; for example, pregnant women are ineligible for treatment with all drugs used for NTDs, and some children are ineligible for some drugs (e.g. children under the age of 9 for doxycycline, etc.) [15], and these individuals continue to be potential reservoirs for infection. Control of the pathogen in human populations can also lead to its relative concentration in less accessible animal reservoirs (e.g. schistosomes in the Philippines, distributed across dogs, horses, pigs and sheep; [15]); or for exclusively animal pathogens, in alternative hosts. A mild variant of rinderpest circulating in wildlife populations became apparent only during the endgame [2]. For FMD, identifying and removing carriers of infection poses a major challenge during the endgame [47,48]. All of these atypical sources of infection may be spatially/temporally clustered, with dynamical repercussions.

### (b) Control measures need to change

#### (i) Surveillance requirements change during different phases of control

Surveillance serves multiple purposes: finding remaining cases of circulating infection; measuring and mapping uptake of vaccine or drugs; detecting emergence of resistance; and identifying populations at risk. Identifying the remaining pockets of susceptible individuals is essential for focusing control efforts, but the spatial distribution and demographic characteristics of these individuals may have altered by the endgame [23]. Ideally, susceptibles could be identified by population-wide sero-surveys, but this is both practically difficult and costly. For human infections, those who are ineligible for treatment must be closely monitored and given alternative preventative measures (e.g. bed nets against mosquitoes given for malaria are also helpful in preventing transmission of lymphatic filariasis [13]).

Detecting infection at very low levels is much harder and requires ever more sensitive and accurate detection methods, as does detection of asymptomatic cases and long-term shedders. These individuals may become more important in the endgame, as occurred, for example, in the elimination of polio [49]. Routine surveillance may no longer be sufficient, and new methods may be necessary, such as active case searching, shown to be effective for smallpox [1]. Remaining infections will naturally tend to be in populations overlooked by interventions until this point (remote locations, areas of civil strife, impoverished communities, migratory populations). Local knowledge of communities was exploited for both smallpox and rinderpest through participatory methods. For rinderpest, in particular, this facilitated surveillance in populations otherwise inaccessible to traditional veterinary services [50]. These innovations in surveillance involved little if any new technology, but instead built on thorough investigative epidemiology and local knowledge (from communities and determined fieldworkers) to identify the causes of persistent transmission.

#### (ii) Implementing control measures becomes harder

As the endgame approaches, the remaining foci of infection are likely to be the hardest to reach geographically, medically and socially (in terms of persuading vaccine refusers to get vaccinated [22]). Geographically, the last foci could be in the most remote areas, or in unapproachable terrain owing to war and civil unrest, as is the case with guinea worm [15]. Alternatively, remaining foci could be in the densest conurbations where identifying and reaching susceptible individuals may be logistically demanding. Densely aggregated urban populations often include informal communities in poor areas, weakly documented by censuses or formal registration systems. This makes both estimating vaccine requirements, and achieved levels of coverage difficult, and may lead to overestimation of coverage success, allowing areas to harbour persistent transmission. To achieve elimination for polio in India, researchers drew on local knowledge to develop detailed microplans in Indian cities, identifying pockets of susceptibility as targets for vaccination campaigns [28]. In densely populated areas, control may be further exacerbated if the *R*_0_ of a pathogen increases with population density [51, p. 89]. Indeed, patterns of smallpox incidence were indicative of increasing transmission with population density [52].

Achieving and maintaining high vaccination coverage is key for the elimination of vaccine-preventable diseases. However, most vaccines require refrigeration, and consequently, the requirement for a ‘cold-chain’ infrastructure presents an important operational limitation. For example, for childhood diseases targeted by the extended programme of immunization (EPI), coverage is often lowest in populations that are more remote, and this remains an obstacle to interrupting transmission [53]. The development of thermostable vaccines was a key innovation for reaching remote or otherwise inaccessible populations, critical to both successful eradication programmes (smallpox and rinderpest). Thermostability was also a critical requirement in the development of oral vaccines for vaccinating wildlife against rabies [54].

If reaching people is measured in terms of treatment success, then  complications such as co-infections can reduce the efficacy of control programmes. For example, gut parasites reduce sero-conversion from oral polio vaccine increasing the number of required doses to 6 or 7 in some areas of India [55]. Similarly, for control measures involving chemotherapy, individual heterogeneity in absorbing drugs will affect efficacy.

#### (iii) Costs per case prevented increase

Locating infected individuals and remaining susceptibles becomes increasingly hard and expensive. Costs of scaling-up control and vaccination programmes increase [56]. Control measures must be maintained until the last case is no longer infectious, and therefore costs per case escalate given the long epidemic tail of low incidence that is typical of the endgame (figure 2, polio). The transient dynamics of infection will therefore be key determinants of time to elimination, and associated costs of maintaining control effort. For example, successive oral vaccination campaigns led to massive reductions in the incidence of wildlife rabies across Europe, but disproportionately greater effort was required during the endgame [32].

These escalating costs and effort can provide a motivation to switch strategies from, for example, mass delivery of vaccine or drugs with the aim of achieving herd immunity, to targeted delivery in remaining foci of infection or high-risk locations or populations. While such a switch is potentially cost-saving, it is also very risky if implemented prematurely (rinderpest and FMD both experienced resurgence following premature cessation of vaccination [2,14]). This is more likely if surveillance is inadequate. For diseases where serological surveillance is required, the need to discontinue vaccination so that sero-positives through infection can be differentiated from those that are vaccine-derived, is inherently risky. For both rinderpest [2] and FMD [14], decisions were required to stop vaccinating in order to be able to assess disease-free status.

#### (iv) Waning support

As costs accumulate, donors fatigue. If donors withdraw, then an elimination programme can be jeopardized and there is a risk of a return to endemicity, with potentially damaging events along the way. Responding to re-emergence is particularly expensive, but is vital to prevent incursions from establishing. Dealing with imported polio outbreaks in African countries that were previously free of wild-type poliovirus cost US$ 850 million from 2003 until 2009 [57]. Communities also become less engaged as disease incidence declines, and become correspondingly less invested in control activities, or start actively refusing the vaccine. This can be particularly problematic where communities are facing many other life-threatening infections for which they are receiving comparatively little support, although still receiving treatment for an apparently vanished infection [22].

Once eradication has been achieved, returns on investment are potentially infinite [58], but to secure commitments, elimination programmes typically need to be time-bound, providing a target which all stakeholders work towards. The more obstacles arise during the endgame, and the more prolonged an elimination programme is, the more difficult it becomes to continue to secure financial (and political) support. A shifting timeline is common for elimination programmes, with advocates for polio eradication having recently pushed for an emergency action plan to finally end transmission and prevent further escalation of costs [29]. Ultimately, if targets are not met and diseases remain endemic, then support for future elimination efforts is likely to be even more difficult to solicit.

#### (v) Incentives change during the endgame

Individual and societal incentives differ greatly during the endgame. For an immunizing infection, it might be best for society to vaccinate above the level of herd immunity to eliminate the disease. Society can then reap the benefits of lower infection rates and associated costs of treatment, increased productivity, and even savings from vaccination cessation in the case of global eradication. From an individual perspective, incentives to get vaccinated or to vaccinate your animals [14,31] rapidly diminish as the threat of infection becomes very slim during the endgame. The risks are further reduced because of protection provided by herd immunity [58]. For the individual, risks from adverse effects from vaccination may be perceived to exceed the risk of infection, which additionally contributes to vaccine refusal [22] (see box 3). As the remaining susceptibles will increasingly consist of vaccine refusers, refusal has a disproportionately larger impact during the endgame compared with the middle game. Financing incentives also change during the endgame and it becomes increasingly important to coordinate international and donor financing, as some communities or countries might need more help than others, especially because local health priorities might not include eradication [9,65]. This was the case in Ethiopia, where famine posed a much bigger problem than smallpox circulating in mild, *variola minor*, form [1].

Until a disease is globally eradicated, there is a risk of re-emergence in populations where *R*_E_ is greater than 1 (box 2). Precautions for dealing with this risk depend on the route by which a disease is transmitted and the mobility of populations. The presence of endemic disease in neighbouring regions poses a threat to disease-free countries, and this can incentivize investment to support the efforts of neighbours. This solidarity is evident in the Americas where countries capable of producing surplus vaccine or with greater wealth provide support to countries struggling to eliminate canine rabies [31]. Similarly, for this reason, the EU is financing a 100 km wide cordon sanitaire of oral rabies vaccination, to prevent the spread of fox rabies back into the region [32]. For diseases that can be easily transported long distances (e.g. polio), these risks are more global.

Our ability to successfully overcome these issues is compromised by serious knowledge gaps across an array of infections and contexts. In §3, we introduce some of the major knowledge gaps in infectious disease control.

## 3. Knowledge gaps

### (a) Spatial heterogeneity and connectivity

Heterogeneity and connectivity of populations are key to disease persistence, but are both host/vector- and pathogen-dependent; consequently, characterizing them may be challenging. For example, the remaining foci of infection may be in remote locations where detection is hardest, and may thus remain undiscovered. Even if their location is known, establishing the degree to which these foci are connected to other locations is rarely straightforward, but of great relevance, as weakly connected places may be relatively ineffective at propagating infection, and therefore of less concern for the endgame than better connected locations [20]. The degree to which completeness and evenness is required for control measures (vaccine distribution, mass drug administration or preventive chemotherapy) also depends on the connectedness of populations. For the elimination of rabies, heterogeneity in coverage can be highly problematic, prolonging the duration of an elimination programme [32] and jeopardizing chances of success [66].

Understanding spatial connectivity can also be crucial to identifying the optimal operational unit for control and surveillance. Ideally, these units should reflect epidemiological units, so that, for example, if an outbreak response is required, then it is focused around where the outbreak is expected to occur, and not excessively deployed in areas that are unlikely to be affected (e.g. rivers and mountain ranges act as natural boundaries to the spread of rabies [67]). Likewise, surveillance that does not to some degree reflect epidemiological units is at risk of entirely neglecting some areas and over-sampling others. The related question of the degree to which control efforts may be spatially targeted (e.g. for immunizing infections, focusing on locations above the critical community size (CCS) because those below the CCS will go extinct independently [68]) is of crucial importance where resources are limited, but requires a combination of modelling (box 4) with data that are rarely available.

Box 3. **The role of the media.**

The media plays an important role in disease-elimination efforts, advocating for increased support, disseminating success stories and often acting as an informal source of surveillance information. This role can be very positive but the media also has potential to have hugely detrimental impacts. Bad news travels fast, and rumours can be very damaging when programme success relies on participation of all (or a large percentage of) members of a community [59]. Even though it has since been proven that there are no causal links between use of the mumps-measles-rubella (MMR) vaccine and autism, media reporting of causality lead to loss of faith in vaccination [60]. This has lead to large outbreaks of measles in the UK (the most recent one in Wales affecting huge numbers of 10–18 year olds [61]) indicative of renewed endemic circulation of infection due to declines in herd immunity [62].

Attempts to present a balanced view in the media invariably lead to overexposure of rumours that are not supported by scientific evidence. For vaccines, this has meant an increase in perceived risks of adverse events, leading to vaccine refusal. Likewise, media reporting of propaganda about the safety of vaccines or their misappropriation for political purposes can be far-reaching, such as during the boycott of polio vaccination in Nigeria [63]. Negative reporting can cause donors to pull out, which again was a problem in Nigeria [64], and can be catastrophic if they lead to a loss of community support. Trying to improve coverage after a scare story is always difficult. Premature reporting of potential success may also be detrimental, if it corresponds to a relaxation of control and prevention measures, as occurred in Bali when several newspapers reported that rabies had been eliminated from the island.

The large audience and potential to drive behaviour change, however, means that the media is a very powerful force. Successful programmes work with the media to help ensure the spread of the right messages as part of their operations (e.g. guinea worm). Drawing attention to a disease through the media helps to maintain momentum during a drawn-out endgame, particularly of politicians and donors, whose commitment is integral to programme success.

Once disease has been eliminated from a given region, the mobility of populations and transmissibility of the pathogen determines both the likelihood of re-emergence and the extent of measures needed to prevent or respond to re-emergence [83]. For human pathogens that spread quickly, the risk is global, while for diseases where either the hosts or parasites are not readily transported preventative measures can be more spatially restricted and elimination can be implemented more gradually. However, pathogen-specific research is required to develop tailored action plans.

### (b) The timelines of control and transition to the endgame

Just as the spatial scale of control efforts may be poorly known, the time-scale of control effort may be hard to predict. For both smallpox and rinderpest, the endgame was relatively short (figure 1), although eradication was declared almost a decade after the last case of rinderpest was detected [84]. By contrast, the endgame has been much more prolonged for dracunculiasis and polio (figures 1 and 2), increasing the costs of these programmes (table 1). The length of the endgame is likely to be a complicated function of the underlying biology of the pathogen, the demography of the host(s), the connectedness of affected populations, the speed of roll out of control measures, their efficacy and the capacity for sustained effort, likely to be itself shaped by political agendas and financing. Consequently, it is almost inevitable that it will be hard to establish whether the endgame is realistically achievable within a finite time-horizon.

**Table 1.** Costs of elimination campaigns (ERR, economic rate of return).

disease	time period	cost (in $ million)^a^	benefit–cost ratio	reference
malaria	1957–1975	>2000	17 : 1	[85,86]
smallpox	1967–1979	98 (international) 298 (total)	483 : 1 (international) 159 : 1 (total)	[87,88]
rinderpest	1986–2008	610	studies ongoing (60 highest estimate) 4–16 for Chad	[84,89]
guinea worm	1986–2015	350	29% ERR	[13,90]
polio	1988–2013	9500		[30]

^a^In US dollars of the indicated time period.

Another important aspect of the time-scale of elimination is the duration of transient dynamics (i.e. short-term fluctuations in incidence, that may even exceed previous levels of incidence, but that will settle to an equilibrium). For immunizing infections, transient dynamics may be expressed via susceptible build-up with late-age large outbreaks (i.e. honeymoons). For other pathogens such as helminths with long lifespans that are not vulnerable to control measures, duration of transient dynamics will be shaped by the persistence of adult worms. Anticipating and monitoring these dynamics will be complicated by challenges to surveillance when incidence is at very low levels. Transient dynamics may be amplified by evolution of the pathogens in response to control measures, towards immune escape, drug resistance and, where live vaccines are used, the emergence of vaccine-derived viruses (observed for both rinderpest and polio [2,28]). Knowledge of these time-scales is clearly key, but still profoundly lacking.

The time-scale over which it is appropriate to relax control measures following elimination is another key question; relating to both the question of transient duration and of spatial connectivity (above). Early cessation of control measures could lead to catastrophic re-emergence (as seen for rinderpest [2]), but costs of continued control are difficult to justify to funders, when disease has not been seen for sometime. International organizations (WHO, OIE) may specify periods for maintained control with no detected cases for certification of freedom, but there has been little research on optimizing these targets. Furthermore, should eradication succeed, post-elimination measures ought to be in place to minimize the risk of re-emergence of the same or a related pathogen that could invade a newly vacated niche [91], but this is an even more uncertain area.

It is very hard to anticipate how long investment and energy can realistically be sustained relative to the time needed to achieve elimination (see range of costs in table 1). Clear goals and deadlines for eradication are especially important, as a prolonged endgame leads to donor fatigue; but these are complicated for all the reasons detailed above. Many target deadlines become moving targets, with potentially negative repercussions.

### (c) Unanticipated immunological hurdles

A final area dominated by knowledge gaps is the degree to which unanticipated immunological hurdles to elimination may arise. For example, for infections where boosting of immunity is occurring via natural infection, this process may not be apparent until the ecology of the pathogen has been radically altered (e.g. as happened for pertussis [40]). For pathogens such as malaria, where low immunity is linked to the most severe infections [92], and frequent re-exposure maintains high immunity, control measures may counterintuitively lead to an increase in adverse outcomes. These outcomes may only become apparent when control measures have been so effective that some portions of the population have very weak immunity, or that remaining cases are linked to immunosuppressing infections, with all the consequent challenges in terms of reservoirs, etc. (see above). This is exemplified by the situation in India, where despite repeat campaigns with some children receiving more than 20 doses of polio vaccine, transmission still occurred. To solve this, a switch to a more immunologically effective monovalent vaccine was required [28,55]. The switch was achieved through an unusual investment in vaccine development addressing safety and ethical requirements to fast track the deployment of the new vaccine.

Immunity may cease to be effective across populations as a whole if vaccine/immunological escape occurs (the time-scales of this are also very hard to anticipate). Where cross-immunity is repressing a co-infecting pathogen, elimination of one pathogen may release the other, with potentially negative consequences if the second pathogen is more problematic (note that this may also occur through relaxation of exclusion via resource-mediated competition) [91].

The implications of the endgame issues and knowledge gaps described above all play out in the context of the socio-economic and political settings in which elimination efforts are deployed. In §4, we provide an overview of how these settings have changed, and have influenced the implementation of elimination programmes and their outcomes.

## 4. Implementation of elimination efforts and health systems

One of the major criticisms of elimination programmes, also referred to as ‘vertical programmes’ is that they selectively target and prioritize a specific issue sometimes at the expense of comprehensive primary care. For human infections, horizontal programmes, by contrast, focus on strengthening primary care and providing ‘health for all’, where selection and prioritization of interventions are shaped by a country's health situations and available government funding. For animal infections, horizontal programmes are equivalent to general strengthening of veterinary services without focusing on a specific pathogen. For countries with weak horizontal programmes or veterinary services, elimination efforts may provide an opportunity to strengthen primary healthcare by integrating their benefits such as training of personnel, improved infrastructure and surveillance capacity including access to diagnostic laboratories. For example, the yellow fever programme developed the first nationwide administrative health systems in many countries, the yaws eradication initiative provided many basic health services and improved national surveillance, the smallpox programme trained an international cadre of health officials in epidemiology and surveillance and served as a basis for EPI globally [9], whereas the polio programme established a global laboratory network that can be used for other health initiatives [93].

Despite these benefits to primary care, the polio eradication programme has been criticized for draining scarce resources of countries where polio initially was not a priority, such as India. For every dollar of foreign funding originally invested in polio elimination in India, the country has spent a hundred dollars more, totalling US$ 2.5 billion, which is more than what the USA has spent on polio eradication worldwide [94]. India has now been polio-free for over a year, which is an extraordinary achievement that took an enormous amount of effort. However, non-polio acute flaccid paralysis (AFP), which has twice the fatality rate of wild polio, and other enteric infections remain a problem [94]. Investing part of that sum into improving sanitation and access to potable water could have prevented some of the non-polio AFP cases as well as many other enteroviruses and gastrointestinal infections spread by the faecal–oral route.

While eradication programmes require a ‘vertical approach’ in terms of targeted disease control, clear goals and deadlines, they can and should be integrated in current health systems and involve local communities (as was the case with smallpox and rinderpest) rather than having a ‘monolithic’ approach with a separate cadre of full-time workers (malaria eradication programme 1955–1978). Elimination programmes also need to be flexible, and recognize that strategies may need to be updated depending on ongoing research and fieldwork, local demography and pathogen or host changing ecologies. The rigid, ‘one size fits all’ approach failed in malaria [95].

Since the era of smallpox eradication, funding for elimination programmes has greatly changed. In the 1960s, funding was mostly provided by the WHO and the nation states. Today, WHO largely has a regulatory role, whereas funding is sourced by many non-governmental organizations, the World Bank and charitable foundations such as the Bill and Melinda Gates Foundation, the Carter Center, Rotary International and global alliances of these partners (e.g. GAVI Alliance, formerly the Global Alliance for Vaccines and Immunization). While government funding is subject to prioritizing among multiple interventions, foundations often fund a specific intervention directly and vertically. These contrasting routes could potentially lead to diffusion of responsibility, and neglected issues are likely to remain unfunded and untreated. This is particularly true for developing countries dependent on technical and financial support from external donors [95]. The millennium development goals (MDGs) have provided further incentives to eliminate diseases associated with poverty and childhood mortality and have provided some coordination amongst goals; however, considerable variability remains.

Box 4. **The role of modelling.**

Application of mathematical methods in epidemiology dates back to 1760 and the seminal paper by Daniel Bernoulli that investigates the age-specific impact of smallpox inoculation [69]. A century and a half later, Ronald Ross’ mathematical models of malaria [70–72] and Kermack & McKendrick's compartmental models [73–75] lay the foundations for the design and quantitative evaluation of control strategies, in particular, conditions for elimination (see critical eradication threshold and definitions for *R*_0_ and *R*_E_ in box 2). This was followed by the development of an array of economic models.

From an economic perspective, optimal strategies will minimize the combined costs of disease burden and control measures; elimination may not always be the best outcome (see Barrett [58] for a detailed overview of economics of eradication). Depending on local epidemiology and economic constraints, the optimal strategy can range anywhere from no intervention to elimination [65]. Cost–benefit analyses of specific country control programmes generally compare a small number of different strategies (not necessarily focusing on the optimal strategy), partly as a result of the difficulty of effectively evaluating the relevant costs [76]. Data are rarely available to define the precise functional form of infection costs or how exactly costs of control scale with control intensity (fig. 4 in Freuling *et al*. [32] illustrates these difficulties). Estimating economic feasibility of eradication programmes requires extrapolating these local cost-effectiveness analyses globally (see table 1 for overview of estimated cost-effectiveness of certain elimination programmes). Additionally, such global extrapolations are often based on data from a handful of countries [77] introducing a potential bias. In the absence of data, strategic modelling has been essential in guiding an overview of key issues.

Data-driven modelling analyses have been deployed to guide policy adapted to local heterogeneities (degree of seasonality in transmission, human demography, etc.) for a range of infections (FMD [78], measles outbreak response [79], designing vaccination strategies [80]). In the context of the endgame, there has been considerable data-driven modelling to predict the impacts of control programmes [34,38,81], or the impact of climate change on disease transmission and distribution [82]. This exercise is often data-limited (see §3). For example, while models suggest that it might be possible to not vaccinate areas below the CCS for immunizing vaccines [68], this will depend crucially on the degree to which these locations are connected to the rest of the population, which is very rarely known. Modelling may still be useful here, as it allows exploration of knowledge gaps, and may inform data collection necessary to close these gaps [24].

## 5. Discussion

There are a number of prerequisites to the successful eradication of a disease. Elimination needs to be biologically feasible (e.g. no unknown or inaccessible animal reservoirs, the life cycle of the pathogen can be interrupted, there are clear clinical manifestations or laboratory tests to confirm cases, and limited environmental persistence [96]). Effective control measures need to exist (such as strongly immunizing vaccines, highly efficacious drugs or simple behaviour changes) and they must be long acting and cost effective. Even where all these conditions are met, obstacles will emerge during the endgame.

It is of paramount importance that the coverage and completeness of control measures required to eliminate the last foci be achievable. The most inaccessible people and places cannot be ignored as these are likely the last strongholds of infection. Levels of surveillance sufficient to monitor disease during earlier phases of control may no longer detect infections circulating at very low incidence. This creates a risk of premature discontinuation of control measures and expensive re-emergence; yet, the costs of prolonged efforts may be prohibitive and draining. Novel surveillance and control tools and strategies can make crucial contributions. Historically, these have included improved vaccines, treatments and delivery mechanisms (thermostable vaccines, monovalent formulations, bifurcated needles, bait delivery, measuring sticks, donated drugs, containment vaccination strategies), and better detection methods (rapid diagnostic tests ([50]; AFP laboratory networks; markers of bait uptake [54]) often capitalizing on local knowledge and communications networks for surveillance and monitoring.

However, none of these innovations matter in the absence of political will and financial commitment—and commitment across a whole spectrum of scales, from local religious leaders through to government officials and global decision-makers. Without political commitment, funding and human resources will be lacking [14], as well as the capacity to engage communities to participate. Elimination programmes have been able to command special attention in the political arena, including the negotiation of localized ceasefires in order to reach vulnerable children [64]. However, mishaps during the endgame may shake both public and political confidence; and perceptions of conflicting geopolitical agendas may further complicate this. Recent violence directed towards healthcare workers in the last remaining countries with continued polio transmission poses a dilemma [29].

It is likely that even with the ideal configuration (a highly efficacious, cost-effective control measure in a context of political commitment), the time-horizon for eradication will be limited to a rather narrow window of opportunity—with the upper boundaries on the window being set by, for example, the risk of emergence of resistance, or evolution of higher virulence, or loss of political momentum, and escalating costs (the last miles tend to cost more).

As we move towards local elimination for a diverse group of infections, it is of value to note that eradication may not always be the best option, particularly given the potential of some pathogens to re-emerge with dire consequences. In an era where pathogens such as polio can be easily synthesized in a laboratory [97], it may be necessary to continue vaccinating after elimination, lowering the economic feasibility of elimination [58,65]. The sustainability of control versus the extended benefits of eradication should be a key research focus. For some diseases, in some circumstances, eradication may not be worth trying. Global eradication may not currently be feasible for measles due to its high *R*_0_, but local elimination and continued control are extremely valuable, as reductions in morbidity and mortality justify the continued maintenance of childhood vaccinations (see also [23]). The same argument holds for massive reductions in the burden of disease achieved for example through chemotherapy for the control of helminths. These results should be viewed as extremely beneficial, rather than as a failure, because elimination has not been achieved. Long-term control might be a more viable option to reduce cases and prevent re-emergence especially if there is no feasible post-eradication strategy.

Nonetheless, thanks to organization, innovation and determination, considerable progress has been made in the global eradication and regional elimination of pathogens and parasites that threaten public and animal health. While the last mile is often the longest, it can also bring the greatest benefits.

## Endnotes

1 Note that although transmission of SARS has been successfully interrupted, SARS does not fit this definition of eradication, as it never became established in the human population. Control of emerging infections differs from control of long-established pathogens and greatly relies on rigorous surveillance and prompt reporting, to guide control methods that prevent initial pathogen spread (e.g. quarantine proved very effective for SARS, but would not be feasible in endemic populations).

2 Yaws is caused by a spirochete *Treponema pallidum*, related to the one that causes syphilis; WHO recently introduced a third target for eradication, in addition to polio and guinea worm, and launched a new initiative to eradicate yaws by 2020 as one oral dose of azithromycin is as effective as intramuscular penicillin [5].
